# Dr. James Taylor’s Introductory Address

**Published:** 1852-01

**Authors:** 


### DENTAL REGISTER OF THE WEST.

Vol. V. JANUARY, 1852. No. 2.

### Original Communications.

Ohio College of Dental Surgery,
November 17th, 1851.

Prof. J. Taylor—Dear Sir: As a Committee of the members of the Class, we respectfully solicit the publication, in the next number of the Dental Register, of the very able and interesting "Introductory," delivered by you in the College, Nov. 3d, 1851.

GEO. L. PAINE,
J. C. WHINERY,
JOS. H. OLDS.

Cincinnati, Nov. 18th, 1851.

Messrs. G. L. Paine, J. C. Whinery, and Jos. H. Olds—Gentlemen: Agreeable to your request, I respectfully submit, for publication, my Introductory.

JAS. TAYLOR.

### INTRODUCTORY ADDRESS

*Of James Taylor, M. D., before the Ohio College of Dental Surgery, Nov. 3d, 1851.*

Gentlemen—We are again permitted, by a kind Providence, to open these halls to the student of Dental Science. How rapidly passes away the few months which intervene between the close of one session and the opening of another.

Life is made up of repetitions—with here and there a novelty—a new phase in the "drama," giving zest to our journey, and stamping indelibly on the mind a few mementoes by which we can retrace our progress.

It is but as yesterday, and these halls were dedicated to Dental Science, and yet we now commence the seventh session.

This enterprise—that of founding a Dental College—is not one of mere speculation, or even designed to be one that would remunerate for time spent and money expended. No scheme lies hidden here for money's gain—no search for that stone which shall transmute all metals into gold; nor ease nor comfort here is sought, except indeed to those who find enjoyment in toiling up the hill of science. No soft, luxurious couch invites the lounger here to calm repose; no royal road to knowledge here is found—naught but determined perseverance holds out hope of gain, of triumph and success.

> This simply is a dental plan
> To elevate the moral man;
> To draw from Science every truth
> That may assist the dental youth.

Permit me, then, on this occasion, to refer to the origin and organization of this Institution, and somewhat bring up its history to the present time. The profession has, for many years, felt the necessity of some systematic plan of instruction—some course which should secure a dental standard of attainment. We had been looking to the medical faculty for years, hoping they would take up this subject and meet the wants of the public, and the dental profession, in this matter. But their course of instruction was already as prolonged and complex as a four months' course would permit, and the idea of uniting the duties of the general practitioner and dentist, was found to be Utopian in the extreme. Either, if well pursued, will tax the talents of any man, and it was rightly supposed that the amount of instruction which might be collaterally given in a medical course, would not be such as would make good operative Dental Surgeons.

The subject of a Dental Chair we know was entertained in several of our medical schools, and the time for instruction, and patients to be demonstrated upon, were obstacles which could not be well overcome.

We will do the medical profession the justice to say, that they have not been indifferent to the need of proper dental instruction, nor to the necessity of a thorough Medico-Dental education; yet, while feeling this need, they have justly felt it due *the Dental* Profession, to act in this matter; and we are glad to say that the faculty of the Medical College of Ohio, on consultation, advised the establishment of this Institution, and that thus far the medical profession has been not behind the dental in its support.

This condition of things existed some eight years since, and on consultation with Profs. Cook and Rogers, both then practising dentists of this city, we determined to apply to the legislature for a charter for this school, hoping to afford in future the student of Dental Surgery the same facilities for knowledge as is enjoyed by those studying medicine.*

We will state more in detail some of the reasons which induced us to make this application.

We found it to be utterly impracticable to induce, as a general rule, our students to enter Medical Colleges, that they might acquire that knowledge of the system and of disease in general, which we conceive to be essential to the skillful operator. It appeared to them, and it really is, out of place to go to those for knowledge in a profession which they do not pretend to practice or teach. But seldom do our best practitioners of medicine, (except when necessity compels,) perform the most ordinary operations in Dental Surgery. Few, if any, prepare themselves with the necessary instruments; and we would here remark, that it is strange that those living in the country and doing a country practice, when they are called on to perform the simple operation of extraction fifty times to any other, yet

---

*It is proper and due to Prof. Allen to state, that after application had been made for the charter, he met Dr. Dogers at Columbus, and they agreed to a change in the charter—placing the Institution in the hands of a board of trustees, instead of the faculty. We think this change a good one.

even here they take more pains to have a good set of amputating instruments than extracting.

The want of proper instruments causes many failures, and these repeated failures drive away their patients, and finally give them a dislike to the operation. To succeed, they *must* have good instruments, and handle them sufficiently often to acquire confidence, precision, and dexterity.

But few, if any of our best practitioners of Dental Surgery, but will acknowledge the necessity for a more thorough course of instruction than has been generally given our students. All such know the difficulties in the way of imparting to their students *real* practical knowledge out of the laboratory. Their own time is occupied at their chair, and but few indeed of their patients will consent to be operated on by *proxy*.

Experience has, we think, fully demonstrated that a standard of dental attainment could not be established so long as instruction was entirely in the hands of private preceptors. Even the term allotted for pupilage could not be determined on or fixed.

Each feel a right to extend or diminish the term of study, to suit their own or their pupil's convenience. Hence we meet in practice those who have studied for years, others for months, and some for only weeks—perhaps *some not at all.*

He who has acquired the art—for with him it is nothing else—in a few weeks or months, and that under an indifferent practitioner, feels that with his natural smartness *he* can impart the requisite knowledge to one as shrewd as himself in half that time. This is not mere speculation. Often, very often, has all this been realized, and sober truth demands the record on the page of dental history. Perhaps no profession has been so utterly bored to death—aye, cursed—with *natural geniuses* and natural mechanics, as that of Dental Surgery.*

---

*A mere taste for mechanic arts is no evidence of dental qualifications; yet how often do we find this urged by the public, and many in the profession, as the great essential for success in dental practice. This is entirely too much relied on, and the effect is to reduce Dentistry to a mechanic art; whereas, no

But the evil ends not here, for while some feel it important and requisite that they demand of their students a course of study which shall embrace anatomy, physiology, pathology, therapeutics, chemistry, &c., &c. Others, having never studied these branches themselves, regard them as perfectly useless, and therefore only teach a little of mechanical dentistry. Hence it has been, with some, difficult to determine whether a dentist was a doctor or carpenter—whether he had been thrust on the world from a physician's office, or from the laboratory of the silver and goldsmith—whether he had been ground over in the mortar of Esculapius, or purified in the furnace of Vulcan. It has been hard to give him a name and character, and this has been the result of the very uncertain course of study generally required.

These truths, forced upon us by observation, impelled us to action; for years of practice strengthened the conviction in our minds, that a dentist as much required anatomical and medical knowledge as the general surgeon. Either would be culpable for ignorance in relation to the parts on which he operates. Duty, therefore, to the public, as well as the profession we had adopted, appeared to demand that a remedy for these evils be put in operation.

There is much of that knowledge which is requisite to the successful and accomplished dental operator, not very easily obtained in a dental office, or of a private preceptor. Besides, those qualified to teach have but little time generally to attend to students.

We wish to be understood; we do not disparage private tuition—on the contrary, we would urge it upon every one entering the profession, and hope soon to see the day when it will be as customary with our dental students as it is with the medical, to enter the office of a preceptor, and continue their

---

branch of surgical science really requires more thorough knowledge, in all those branches of science now taught in our dental schools. Without talent and energy to investigate and understand these, eminence need never be expected in the profession.

pupilage at least as long. They have as much to learn, for what they dispense with on the one hand they must take up on the other. The whole range of mechanical dentistry, which no man can perfect himself in, in one year, is an addition to the studies of the medical student, and really not necessary to the medical practitioner. Yet tell us what part of his knowledge is useless to the dental practitioner? True, he may not wish to turn accoucher, yet there is much connected with gestation, lactation, and diseases of females and children, which should interest him very much, and which he should not be ignorant of.

Are medical schools *aids* to the student of medicine? Do they afford facilities for the acquisition of knowledge? Their multiplicity would appear to answer in the affirmative. If they are necessary, then dental institutions are equally so; for no argument in favor of the one can be made without it will apply also to the other. Their studies are in a great measure identified. The principles on which practice is based are identical; they are one and the same science, having in view the alleviation of disease and restoration to health.

That ultimately dental schools will become as important to dentists as medical schools to physicians, we have no doubt; both alike rest on the nature of the studies to be prosecuted.

These studies are such that to be enforced they require more or less demonstration. This is particularly so in elucidation of that which pertains to dentistry, for here we have science and art combined, as indeed is the case in all which appertains to general operative surgery.

We would further remark, in relation to the causes which determined us in the establishment of this school, that this great valley of the west, now the center instead of the border, fills not up more rapidly with her thousands upon thousands of energetic and industrious population, filling all the other avocations of life, than increases also in equal ratio the number of her dentists.

Need we say that the same spirit of determined perseverance in improvement, and effort to excel, distinguishes no avocation to a greater degree than the dental. There cannot be shown a pursuit in life which has in the last twenty years so extensively increased its capacities for doing good. The most of those most instrumental in effecting the change, particularly in the west, yet survive to rejoice in the brightening prospects of our science.

One star early rose—shone with peculiar brightness—did much to dispel the darkness of ignorance and superstition which then existed, and went out in the meridian of his splendor—Dr. Ratrie, of Lexington, Ky., deceased about twenty-two years since. He died young, yet lived long enough to gain a reputation which will ever be enviable.

If the east can boast a Hudson, a Gardette, and a Hayden, the west can with pride claim a Ratrie and a Putnam.

But the fact cannot be denied, that but few attain the same professional excellence. But few, perhaps, appreciate so well the necessity of a thorough preparation. Most *tug* on, hoping here and there to gain an idea which may be of service in practice. They commence not half prepared, and die before the other is accomplished; yet, during the term of their pracice they are gaining all the knowledge they can from their suffering patients.

The desire to see that pursuit in which we are engaged prosper, and attain in public estimation a character and name for usefulness, is very laudable. The man who is devoid of professional pride, is ready for any species of charlatanism which will bring him gain, without, indeed, wounding the first feeling of his professional standing. His whole soul is wrapped up in self, and to him the mighty dollar attracts every spark of honorable merit. This desire of excellence, when in connection with a pursuit which is essential to our health and comfort, should be the more zealously cherished. The real importance of any pursuit should be in proportion to its usefulness.

We have spread out before us the largest, the most productive and delightful valleys of the world—bounded on the north by the lakes, on the south by the Rio Grande, on the east by the Alleghanies, and west by the illimitable far west, stretching to the heights of the Rocky Mountains.

This great garden spot of the world is filling up with millions of freemen, a large majority of whom can pay for and will demand dental operations. As this mighty central power strides on to greatness, and grandeur, we see every profession and every pursuit in life putting forth the energy of youthful ambition to secure an influence and name in the page of that history which must record the deeds of all.

Shall dentistry, as a science, find on this page no name? no place of record or of praise? We feel that such should not and *shall* not be the case; and if the written scroll of man's record should be lost in the future, that the sepultures of the departed and forgotten shall show by the imperishable evidences of our skill the importance of dental science.

Need we remark, that with these views and feelings our application to the legislature anticipated success. We found Ohio, through her legislature, willing to extend every facility for the promotion of a science so essential to the health and well being of the public. Hence, chartered privileges were conferred on a board of trustees, who, anxious to further the objects alluded to, met at as early a day as practicable, and organized this school. The faculty thus empowered to act in a public capacity, being shielded by law and sustained by a liberal and intelligent board of trustees, have spared no pains or personal effort to secure to the profession as many advantages for the developing and building up of dental science as is possessed by the medical.

Circumstances have been such that a permanent faculty, which should co-operate with each other for six or eight years, could not be obtained. Profs. Cook and Rogers retired from practice. The former retained his professorship only two years, when his removal to the country rendered it necessary to resign. The latter retained his position in the school for three years, when his engagements became such that he could not attend to his college duties any longer, and he felt it his duty to resign. The chair of anatomy, after Dr. Cook's resignation, was one winter filled by Dr. Potter, then for two sessions by Dr. Shotwell of the Medical College of Ohio. The chair of operative and mechanical dentistry was vacated last winter during the session by Dr. Leslie. These circumstances were truly discouraging, but it did not render the necessity for a dental school less imperative. It appeared, indeed, to render it important that a more general interest should be taken in the cause of dental education, that the profession should be identified with the institution.

The faculty had induced a gentleman devoted to education, Mr. Talbot, to erect this building with special reference to the wants of this school, and had leased it for ten years, with the privilege of purchase at the stipulated price of $3,400.

To make the institution permanent, with a fixed location for a dental and anatomical museum, it appeared to be necessary that a building of some kind should be set apart for that purpose, and none other in the city was so well calculated for the purpose as this; and further, the price of real estate having advanced since this was leased, and a price fixed upon it, none other so eligible could be obtained so cheap.

About eighteen months since, after having well weighed these circumstances in our minds, we determined to address some of our leading dentists on the subject of a purchase of this house and lot, with the permanent fixtures belonging to the institution, including the anatomical preparations which belonged to the professor of anatomy. These fixtures and anatomical preparations were priced for this purpose by the faculty at $400.

Dr. Wood gave for the anatomical museum $250, which he liberally put in as two shares of stock at $100 per share. The other fixtures cost some $300 or $400, and was also put in as two hundred dollars, or two shares. The whole has thus been divided into thirty-eight shares of $100 each, and we are glad to say to the profession that the stock has been so nearly taken that the purchase has been made, and we this day open this, the seventh annual course of lectures, in the Ohio College of Dental Surgery, in this hall, belonging to the profession, and set apart by them for the purposes of dental education.

This noble conduct on the part of the profession needs no commendation from us. The simple announcement of the fact speaks volumes, and is better evidence of the estimation with which the profession regard the subject of education' and the need of this institution, than all the labored eulogies which could be written on the subject.

We would take this opportunity to allude to the kind manner with which the profession have entertained this subject, and responded to the letters which we have addressed them. Many necessarily felt obliged to decline taking stock, but we are glad to say, that but in one or two instances has this been done from any feelings of hostility to the school. In almost every instance the response has been favorable to the plan, and although many such could not, from inability, take stock, yet they have encouraged us in the prosecution of the enterprise.

We would also make honorable mention of the fact that so many have not forgotten their alma mater, but have, with most commendable alacrity, came forward and done all and more than could have been expected from them.

The graduates and students of the school have taken some twelve shares of stock, and this has been subscribed for by as many individuals. The object has been to have the stock taken by as many different subscribers as possible. The subscribers may ultimately divide the stock and make twice as many shares, and thus afford more of the profession an opportunity to become subscribers. We wish all the profession who have really a deep interest in the advancement of dental science, to co-operate with us in the effort to secure a thorough medico-dental education, for the future doctors of dental surgery.

The stockholders are expected to select the different members of the faculty, and nominate them to the board of trustees for appointment to the different chairs in this school; and they have also the right to determine the period of study, the qualifications required of the graduate, and, indeed, the general supervision of the course of instruction. Also, to appoint a a committee whose duty it shall be to examine, in connection with the faculty, all candidates for graduation.

Acting on this plan, they have selected the present faculty, who have been appointed by the board of trustees, and now commence their duties under a new order of things, which, we are glad to say, has promise of future prosperity and good to the profession.

We hope soon to see a library, anatomical and dental museum in connection with this institution, which shall be an honor and a pride to the profession; and at each annual commencement a convocation of the profession which shall be profitable and interesting, leading to an exchange of thought and a unity of purpose, which cannot fail to advance the cause for which we labor.

It could not be expected that a new institution of this kind should at once spring up and present itself perfect in every part. We have had but little to guide us in the best measures to be pursued. This, so far as we know, is the second chartered school of dental surgery in the world, and when this went into operation, the Baltimore school had not been long established; so that we could not derive much benefit from their experience. Our object, however, has ever been to meet the wants of the profession, and afford every facility for the aquisition of dental knowledge.

It takes years for an institution of this kind to become generally known ; besides, the prejudice of some and the indifference of others, are to be overcome; and time only can effect this, backed by a conscientious and faithful discharge of duty.

Need we speak of opposition, silent, secret, and watchful? Are not all, is not every member of the profession, every one who suffers from disease of their dental organs, interested in every effort to combat disease and afford relief to suffering humanity?

It would appear scarcely possible that a selfish feeling could here find cause for opposition—that true professional merit should ever fear that which should elevate the general standard of the profession—and *true* merit does not. But there are ever those who are jealous and discontented—who never find things going right—everything is out of sorts—the off horse should be the leader, and the leader should be hitched to the tail end of the wagon. But such is human nature in its unregenerate state, and we should go steadily on, regardless of all such trivial causes for discouragement.

Yet it is strange, and sometimes amusing, to see what trouble and fibbing such will sometimes go, to accomplish an object. The truth is far more easily told, for it requires no invention. But we forget, the profession have a name for invention.

Let us see the caricature, first and foremost. Two doctors of medicine, and the same number of dentists, spend four months in lecturing to one student; yes, only one, and this is sent on the wings of the wind from New York to Nashville, or rather from this to New York and also to Nashville, how much farther north and south we know not, for we have not taken the pains or trouble to trace the *lie* any farther, or to even find the *pet's* loving *daddy*.

Need we speak of students dissuaded from attending lectures, after a long journey for that purpose. Yet such is the case and but that we are giving a history of the school, we should not even allude to such contemptible conduct.

We believe that the books of the institution show the lamentable fact that the faculty *did*, for two sessions, lecture to seven students each session, and they did it with the full weight of their responsibility resting upon them. They did it feeling that the cause was too important to be discarded or abandoned, if they could get seven or even five to devote their time and energies to properly prepare themselves to practice a profession of so much importance. But these, gentlemen, were the dark days, and we knew that circumstances over which we had no control caused this; that the "fell destroyer which swept over the land with the besom of destruction," and the late and unavoidable re-organization of the faculty, could not be other than unpropitious.

They did it, however, in fulfillment of a duty which they had assumed, and who would suppose that conscience does not now whisper an approval?

We rejoice, gentlemen, that we have co-laborers who have "been proved and not found wanting," who are willing to labor in this good cause, and who feel that their best remuneration is advancement of science and the good of the student.

We conceive that the necessity for an institution which shall have for its object correct dental education, needs no argument to make it apparent to every reflecting mind, and that no location more demands it than the Queen City of the west. The fact that the means of education should be co-extensive with the population and wants of the community, all must admit. It is much easier to prevent an evil by proper and early education, than to correct that evil after it has taken deep root and acquired strength by familiar association. These facts apply with as much force to the profession and practice of dentistry as to anything else.

We are then called upon to determine, and I mean by we, *all* engaged in the practice of dentistry, whether our science shall, or shall not grow with the growth and strengthen with the strength of the swelling tide that must soon fill up this great valley.

Time must determine this, but we are free to confess that we anticipate the ultimate success of a dental school of the kind now in operation. True, others may soon be called to perform those duties which we, in our feeble capacity, try to discharge. This, however, will not alter the demands of the public or the duties which attach to the instructors.

As we have thought proper to give a short history of the necessity for, and organization of, the Ohio College of Dental Surgery, permit us here to add, that although called into being by the State authorities, yet no fund has been afforded us by her liberality, no donation made, but individual effort alone has reared this structure and placed within its walls all the means for instruction we now possess. We have gone on adding yearly that which we see appropriate and needful, hoping, as time advances, our library and museum shall increase, till all that can instruct shall be accessible to the student.

We would, therefore, repeat, that it affords us great pleasure, as each year renews our labors, to see, on occasions like the present, not only those who come for instruction, but also those who feel a general interest in the cause of science.

Some of you cheered us with your presence, your sympathy, and kind attention during last session. To such, and those who now first take seats in this hall of science, we bid you a hearty welcome, and hope, as we press on in the same path of scientific research, that we shall find much to instruct and amuse, much to elevate, to expand and enlarge our minds and hearts; and as unity of purpose begets unity of feeling, may we become as one in the great effort to advance our favorite science.

The occasion is calculated to stir up feelings which have, for a time, lain inactive—to throw back the mind on the past, and fix it on many scenes yet vivid with delightful instruction. The casual meeting of a friend, whose meanderings in life have kept us apart for months or years, and who at last, with ourselves, has been attracted to the same spot, thrill us with delight, and although our paths again diverge with only a moment's greeting, yet the feeling of pleasure is impressed on the mind, is reflected in the face, and dies not away until repeated collision with other objects gradually hides from view; but the impression is not entirely erased.

If the meeting of friends afford us pleasure, and each renewal thereof is worthy of remembrance, should not also the spot which brings them to mind be viewed with delight? What friend sticks more close than that knowledge which is founded on truth, and what spot more to be loved than where the truths of science are unfolded? To these truths let us bow, and during the session now before us, make these halls the center of our attractions. Here let us daily meet, and let each visit to this spot be made an occasion of pleasure and profit. Here we meet to enlarge our knowledge, to dive into the arcana of nature's wonderful works, and trace out in beautiful and systematic order the philosophy of diseased as well as healthy action. Should not our hearts expand with our minds, as we see spread out before us the great law which governs our economy, make us feel grateful to that Power and Wisdom which planned and keeps all in order.

Gentlemen of the Class, as we are again about to assume the duties and responsibilities devolving on us, as appointed teachers, in a very important branch of the healing art, permit us to draw your attention for a short time to our relative duties—the duties we owe you as preceptors, and the reciprocal duties involving on you as students.

We do this the more freely in this, our introductory, because, unless we properly appreciate our distinct and separate duties, it is hardly possible that we get along during the session with that harmony and good feeling which is essential to the success of our winter's labors.

It is best, in all the transactions or intercourse of life, that we mutually understand each other. This is necessary to prompt and energetic action. "A house divided against itself cannot stand;" so any organization of individuals divided within themselves, all acting separately, without unanimity and concert, must soon dissolve, or fail in all their efforts to do good.

Concert of action and unity of purpose is essential to success in all undertakings where more than one is necessary to accomplish any given labor.

In all the relations of life, where duty is imposed on the one part, a relative obligation exists on the other. This is essential to the well being and order of society. If we give an obligation for the payment of a hundred dollars, it is your duty to present that for payment; because, so long as you retain or withhold our obligation, duty nor justice requires us to make payment. How preposterous to exact of us to hunt up our note, when it is your duty to keep it, and when due present it. Yet, on first view, how apt are we to think that he who pays the money is the humble servant of the other, and has all the duty to perform. But we find, on examination, that where duty is imposed on one part, an implied obligation is just as binding on the other.

In accepting the appointment as professor in this institution, we have imposed on ourselves a certain and specific duty. This is, to teach that particular department of medical and surgical science which attaches itself to our chair, and when certain requisitions are complied with on the part of the student, we have an additional obligation resting upon us, and this is, to devote a certain portion of our time in giving them such instruction in our branch as our knowledge and capacity may enable us.

But you, gentlemen, like the holder of the note, cannot expect or demand the discharge of our duty unless you present yourselves to receive it at the appointed time and place.

We are not expected to seek you out and force our lessons upon you, but if we come ready to impart what we have, and you are not on hand to receive it, no blame can attach to us, should you never get it in your possession.

Our duty is, properly to teach, and we do not feel at liberty when in the chair, or when respectfully called upon, to withhold anything that may be of service to you in the theory and practice of our profession. But other than this we do not pretend to teach; all else belong to those equally bound to give you information, and as we should hold ourselves culpable for not imparting such information on our particular department as *we* can, so we should hold the assumption of these duties as the heighth of arrogance on our part.

We teach not anatomy or physiology, pathology or therapeutics, but we operate on a structure, demonstrated and philosophized on by one, and described as diseased and physicked by the other. Necessity may, at times, in our illustrations, compel us to speak of the anatomical structure, as well as the diseased condition of the part; yet it should always be in deference to our worthy colleagues, who specially devote themselves to that part of your studies. We regard too much that beautiful order which is essential to the success of all organizations of this kind, to arrogate to ourselves the duty of others, or throw aught in the way of the harmonious action and prosperity of the school.

Those who study most any particular branch of science, should be of it the best teachers.

We consider it our duty, therefore, to teach the practice of dental surgery, to minutely describe every disease of the dental organism and the means of cure, to expose all the errors that may exist, so far as we can prove them to be such, by the presentation of plain and simple truth. We do not consider ourselves bound to give the practice of the emperic and charlatan, only so far as it may be necessary to expose that quackery which stalks abroad throughout the land, professing to do all things, to change the laws of diseased and healthy action, to improve on the perfection of Infinite Wisdom, and with the magic wand of bold effrontery—

> "Relieve our pains with wondrous skill,
> And say to tooth-ache, be thou still."

Or go to work in older style and say—

> "Tooth-ache, tooth-ache go away,
> And from this tooth forever stay."

We do not consider it our duty to tickle your fancy at the expense of truth, to envelope facts in the shroud of mystery, to take you to the clouds in search of the sublime, and wrap your imagination in the mantle of attempted eloquence, and let the simple and plain presentation of truth be sacrificed, or in the least distorted, by any effort to amuse.

We come before you with our science in one hand and truth in the other, and ask you to investigate and then receive them.

Our investigations will lead us to examine the views and practice of particularly those who have long labored in the profession, and who, although "now dead, speaketh," through their written works. They have left us the result of their observation and experience, and many of them have been ornaments to the profession, and deserve much of us. Their works, imperfect as in some respects they now are, yet deserve a fair and candid perusal. We must recollect that they labored without many of the advantages we now possess, and that we have had the full benefit of their experience. We may find, on a retrospect of what *we* have done, that *they* have done more to advance the cause of science than we. We labor to little effect if, in addition to their light, we have that of the present day, and fail to leave our science in a better condition than we found it.

But we have monthly and quarterly thrown upon us from the press the experience of those who now, with us, devote their time and talents to perfect that which has been left us. Many of their views require time to be tried and proven, and it becomes us to weigh well every improvement that may be suggested, and if it be of advantage, at once adopt them.

This is said to be an age of improvement; let us not, however, throw aside that which we know to be good for the mere speculations of theory.

We see that the duties of the teacher are plain and simple, but at the same time arduous and confining. He gives you the result of his experience, and he has to compare that with the experience of others, weigh the results of each, determine that which is best, and recommend it as such to the student. Indeed, he must prove all things it is his duty to teach. In addition to what has been said, let it be borne in mind that there is a specific time when he should meet the class, and there impart the result of his observation and experience.

We will sum up in a few words what we suppose to be the duties of the student. It is understood, gentlemen, that you come here to be instructed; that you view knowledge of a certain kind to be essential to success in that department of medical science you have chosen for your future pursuit in life. The necessity for this knowledge cannot well be overestimated.

"A little knowledge is a dangerous thing,
Drink deep or taste not the Pyerian spring."

Had the poet been a dentist and written the above with reference to his profession, nothing could have been more appropriate.

Ignorance is the bane of all our enjoyments, maring every pleasure and polluting every stream of life.

What is ignorance but a want of knowledge? All error is founded on ignorance. It is, therefore, your duty, gentlemen, to sift truth from error, to search after truth as with a lighted candle, to be always at your place, ready to receive that which is imparted, and willing to believe that which is proven. It is not your duty, however to take mere assertion for fact, but that which is supported by reason and analogy should be relied upon. You will find it impossible to prepare yourselves too well for the duties of your profession; hence, while you are engaged in its study, let this be your only pleasure. The gayety of life, the excitement of fashion, the morbid sentimentality of fiction, and the dreamy reverie of success and fortune, without action, are incompatible with true excellence or professional attainment. To excel, you must study, and to study with a fixed purpose is to excel.

While in this institution, specific studies should engage your undivided attention. These are such as are specially taught by the different professors, and you will find that to do justice to these, and keep up with the lectures delivered, there will be no *time for any* other.

It is hardly to be expected that a lecturer will feel that interest in his subject as to make his lectures interesting and acceptable to his class, unless he has their attention.

Time, gentlemen, will not permit the particular specification of all the duties devolving on us. There are many which courtesy and good feeling will dictate, that need not be alluded to on this occasion. Permit us, however, to add, that at the close of the session another duty is imposed. This is, to examine the qualification of those who shall be candidates for the honors of this institution. The legislature of the State, from whom we derive authority to act, the board of trustees, and the public at large, expect of us to guard well the escutcheon of our profession. It is your privilege, after having complied with the requisitions of the institution, to demand a fair and candid examination, and if found worthy, it is our duty to recommend you to the board of trustees for its highest honors. When we come to close our winter's labors, it will afford us unfeigned pleasure to lay before that board the names of all who may demand it as a reward of merit.

